# Analysis of whole transcriptome reveals the immune response to porcine reproductive and respiratory syndrome virus infection and tylvalosin tartrate treatment in the porcine alveolar macrophages

**DOI:** 10.3389/fimmu.2024.1506371

**Published:** 2025-01-13

**Authors:** Kun Du, Yu Xia, Qian Wu, Miao Yin, Hong Zhao, Xi-wen Chen

**Affiliations:** Animal Disease Prevention and Control and Healthy Breeding Engineering Technology Research Centre, Mianyang Normal University, Mianyang, China

**Keywords:** PRRSV, tylvalosin tartrates, lncRNA, miRNA, anti-inflammation, pig

## Abstract

**Introduction:**

Porcine reproductive and respiratory syndrome virus (PRRSV) is a major pathogen that has caused severe economic losses in the swine industry. Screening key host immune-related genetic factors in the porcine alveolar macrophages (PAMs) is critical to improve the anti-virial ability in pigs.

**Methods:**

In this study, an *in vivo* model was set to evaluate the anti-PRRSV effect of tylvalosin tartrates. Then, strand-specific RNA-sequencing (ssRNA-seq) and miRNA-sequencing (miRNA-seq) were carried out to profile the whole transcriptome of PAMs in the negative control, PRRSV-infected, and tylvalosin tartrates-treatment group.

**Results:**

The ssRNA-seq identified 11740 long non-coding RNAs in PAMs. Based on our attention mechanism-improved graph convolutional network, 41.07% and 28.59% lncRNAs were predicted to be located in the nucleus and cytoplasm, respectively. The miRNA-seq revealed that tylvalosin tartrates-enhanced miRNAs might play roles in regulating angiogenesis and innate immune-related functions, and it rescued the expression of three anti-inflammation miRNAs (*ssc-miR-30a-5p*, *ssc-miR-218-5p*, and *ssc-miR-218*) that were downregulated due to PRRSV infection. The cytoplasmic lncRNAs enhanced by tylvalosin tartrates might form ceRNA networks with miRNAs to regulate PAM chemotaxis. While cytoplasmic lncRNAs that were rescued by tylvalosin tartrates might protect PAMs via efferocytosis-related ceRNA networks. On the other hand, the tylvalosin tartrates-rescued nuclear lncRNAs might negatively regulate T cell apoptosis and bind to key anti-inflammation factor IL37 to protect the lungs by *cis*- and *trans*-regulation.

**Conclusions:**

Our data provides a catalog of key non-coding RNAs in response to PRRSV and tylvalosin tartrates and might enrich the genetic basis for future PRRSV prevention and control.

## 1 Introduction

Porcine reproductive and respiratory syndrome virus (PRRSV) is a major pathogen that has caused severe economic losses in recent decades (1). Pigs are PRRSV’s sole natural host species. The typical host innate or adaptive immune characteristics of PRRSV infection include persistent viremia, a strong inhibition of innate cytokines, dysregulation of NK cell function, rapid induction of non-neutralizing antibodies, delayed appearance of neutralizing antibody, a late and low CD8+ T-cell response, and induction of regulatory T cells (2). Although several modified live vaccines have been designed to prevent PRRSV infection for over two decades, it has achieved little success in the swine industry due to immune escape and high variability of PRRSV. Recent studies have revealed that improving the host’s anti-virial ability presents a promising opportunity to reduce the losses caused by PRRSV (3). Thus, screening key immune-related genetic factors of the host responding to PRRSV is a priority for this emerging strategy.

Non-coding RNAs (ncRNAs), such as microRNAs (miRNA) and long non-coding RNAs (lncRNAs) are important genetic factors that have been widely recorded to regulate the development of complex diseases (4), immune functions (5), and response to drug therapy (6). The PRRSV infection is major restricted to porcine alveolar macrophages (PAMs) (2). Previous studies have revealed that miRNAs were involved in modulating PRRSV replication by directly targeting the PRRSV genome (7), targeting signaling pathways involved in PRRSV replication (8), and targeting host factors involved in PRRSV replication (9) in PAMs. Compared to miRNA, which primarily functions in post-transcription, lncRNA functions in a more complex manner, mainly depending on its subcellular localization (10). LncRNA that reside in the cytoplasm can act as competing endogenous RNAs (ceRNAs), or miRNA sponges, to communicate with each other by competing for miRNA-binding through common miRNA response elements (MREs) (11). In this context, lncRNA interacting with miRNA might be an important mechanism during the anti-PRRSV process of the host. On the other hand, previous studies have revealed that lncRNAs could regulate the expression of target genes in a *cis-* or *trans-*regulation way in the nucleus (12). The *cis-*acting lncRNA *lnc-CAST* could positively regulate the expression of neighboring *CXCL8* through histone H3K27ac, increasing chemokine expression and lung damage during PRRSV infection (13). *Trans-*acting lncRNA *MAHAT* could bind and negatively regulate *ZNF34* expression by recruiting and binding DDX6, increasing type I interferon expression, and decreasing PRRSV replication (14). Nevertheless, in addition to functional studies of a few ncRNAs in PAMs, our understanding of the changes in the whole transcriptome of PAMs during PRRSV infection alleviated by medication remains largely unknown.

Tylvalosin tartrate exhibits a broad-spectrum inhibitory effect on the proliferation of PRRSV both *in vitro* and *in vivo* (15, 16). Consequently, it is routinely employed in pig farming practices as an effective means to mitigate the consequences of secondary bacterial infections and to suppress the replication of PRRSV within pigs. In this study, an *in vivo* model was set to evaluate the anti-PRRSV effect of tylvalosin tartrates. Then, the PAMs were isolated to construct sequencing libraries of the whole transcriptome. We comprehensively determined the changes in mRNAs, lncRNAs, and miRNAs of PAMs under tylvalosin tartrate administration. Furthermore, an optimized RNA subcellular localization classifier was trained to allocate mRNAs and lncRNAs into the cytoplasm and nucleus based on the graph neural network and attention mechanism. Then, we performed functional analysis and screened for key immune-related genetic factors from the cytoplasm or nucleus RNA. Our study is expected to shed new light on the molecular mechanisms of non-coding RNA regulating immune functions in PAMs

## 2 Materials and methods

### 2.1 Ethics approval

All experiments were performed in accordance with relevant guidelines and adhered to the ARRIVE guidelines (https://arriveguidelines.org/) for the reporting of animal experiments. This study was carried out in accordance with the principles of the Basel Declaration and recommendations of the Guide for the Care and Use of Laboratory Animals (http://grants1.nih.gov/grants/olaw/references/phspol.htm). All surgical procedures involving pigs were performed according to the approved protocols of the Biological Studies Animal Care and Use Committee, Sichuan Province, China. The protocol was approved by the ethics committee of Mianyang Normal University under permit No. SKY101368.

### 2.2 Animal experiments and PAM collection

**Figure 1:**
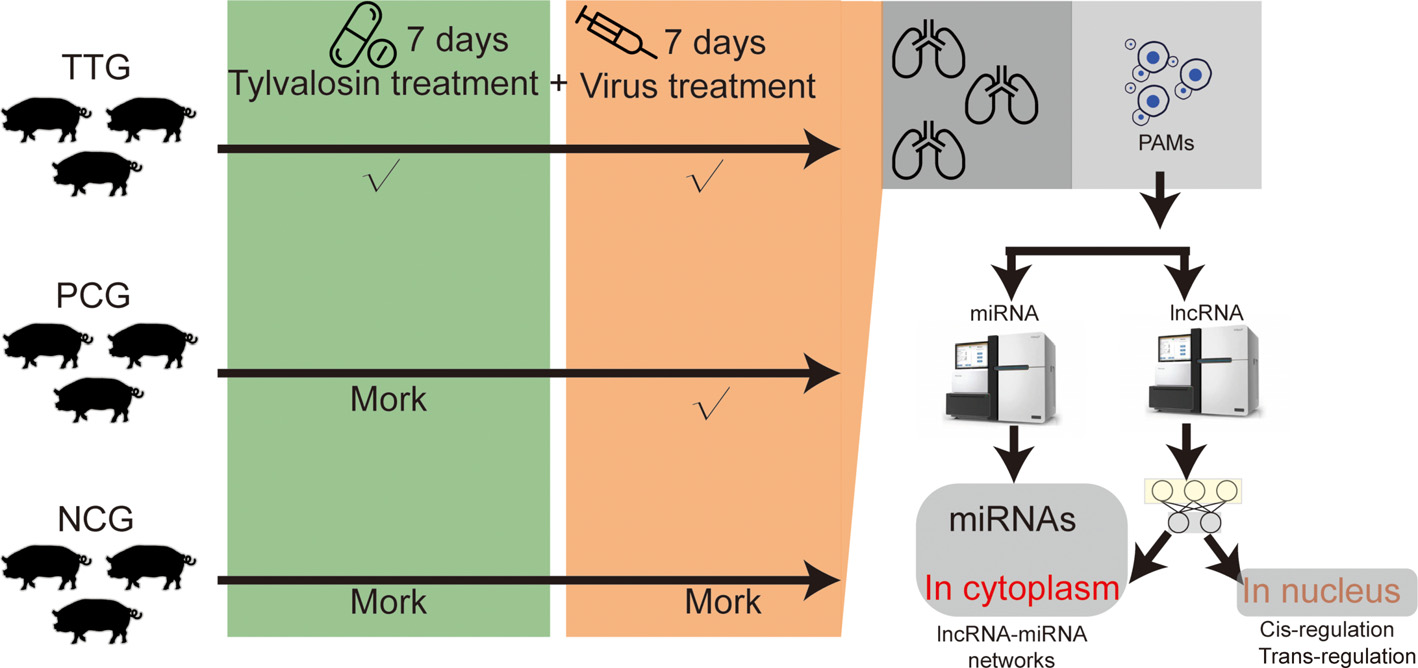
Experimental design in this study.

In this study, tylvalosin tartrate was purchased from ECO-BIOK Animal Health Products Co., Ltd (lot number: 230203, tylvalosin ≥ 85%, Zhejiang, China). The PRRSV was isolated from a pig farm in Mianyang, China, in 2023 (MY-23-1 strain). The virus in the blood sample was isolated and purified in Marc-145 cells. Then, PCR amplification (*NsP2*, *Orf5*, and *Orf7*) and Sanger sequencing were used to type the isolated PRRSV according to our previous study (17). The sequencing data of *Nsp2*, *Orf5*, and *Orf7* were uploaded to the NCBI GeneBank database (https://www.ncbi.nlm.nih.gov/gene/) with an accession number of 2907023. A total of nine pigs (crossbred piglets of Landrace × Large White) obtained from a commercial farm were raised in the animal experiment center of Mianyang Normal University, Mianyang, China. These pigs were weaned 30 days after birth and fed a standard diet and water ad libitum. The pigs were numbered by ear-tagging and acclimatized for 1 week before treatments. These pigs were found to be negative for PRRSV, classical swine fever virus (CSFV), Porcine Circovirus Type 2 (PCV2), and pseudorabies virus (PRV) using commercial ELISA kits (IDEXX, Maine, USA) and were randomly divided into 3 groups, including the Tylvalosin-treated group (TTG, *n* = 3), the positive control group (PCG, *n* = 3), and the negative control group (NCG, *n* = 3). PRRSV is an acute infectious virus that can cause clinical symptoms in piglets within 12 hours. Therefore, we adopted an early medication approach to maintaining a high concentration of tylvalosin tartrate in pigs during the challenge. Before the PRRSV challenge experiment, pigs in the TTG were fed with normal diets supplemented with 20% tylvalosin tartrate (5 mg/kg·bw) for 7 days. The pigs in TTG were fed normal diets without tylvalosin tartrate. The pigs in TTG and PCG were subjected to a challenge with 3 × 10^5^ TCID50 of PRRSV strain MY-23-1, and the pigs in NCG were injected with an equivalent volume of DMEM. On the 7th day after the virus challenge, the pig was slaughtered, and the entire lung tissue was isolated for downstream PAM collection (**Figure 1**). The method of PAM isolation referred to a previous study conducted by Peng and colleagues (18). Briefly, approximately 200mL of PBS was slowly injected into the fresh lung tissue from the trachea, and then lung lobes were gently massaged under sterile conditions. The PBS washing solution was transferred into a centrifuge tube for low-temperature centrifugation at 1000 rpm, and PAMs were collected after removing supernatant fluid. These PAMs were immediately snap-frozen in liquid nitrogen and stored at –80°C until RNA extraction.

### 2.3 Total RNA extraction and strand-specific RNA sequencing

Total RNA was extracted using RNAiso Plus Reagent (TAKARA, Dalian, China) following the manufacturer’s protocol. The purity and integrity of the RNA were determined using Nanodrop 2000 (Thermo Fisher Scientific, Waltham, MA, USA) and Agilent Bioanalyzer 2100 system (Agilent Technologies, CA, USA), respectively, and RNA concentration was measured using a Qubit^®^ RNA Assay Kit and Qubit^®^ 2.0 Fluorometer (Life Technologies, Carlsbad, CA, USA). Only samples with RNA Integrity Number scores > 8 were used for downstream library construction.

A total of approximately 2 μg RNA was used to construct the strand-specific library. The rRNA of total RNA was digested using a Ribo-off rRNA Depletion Kit (Vazyme, Nanjing, China). The RNA The libraries were prepared using the NR603-VAHTS Total RNA-Seq Library Prep Kit (Vazyme, Nanjing, China) following the manufacturer’s recommendation. Briefly, the RNA was fragmented into 100-300 bp lengths. Then, the first strand of cDNA was synthesized using First Strand Enzyme Mix (Vazyme, Nanjing, China). Subsequently, the second strand of cDNA was synthesized using the Maturity Strand Marking buffer (Vazyme, Nanjing, China). The USER enzyme was used to degrade the cDNA strands that contained U base instead of T base. The obtained cDNA was amplified using the high-fidelity polymerase (Sangon Biotech, Shanghai, China). The quality of libraries was checked using a Qubit (Life Technologies, Carlsbad, CA, USA), and all purified libraries were sequenced on an Illumina NovaSeq platform. Finally, the reads with paired-end 150-bp length were generated. The sequencing quality was checked using FastQC software (v0.12.1) (19). The reads with N base number > 5 and mean quality < 15 were recorded as low-quality reads. The sequencing adapters and low-quality reads were removed using Fastp software (v0.23.2) (20) with parameters of “–detect_adapter_for_pe”, “–n_base_limit 5”, “–cut_mean_quality 15”.

### 2.4 *De novo* reconstruction of transcriptome and lncRNA identification

The clean reads were mapped to the pig genome (susScr11, retrieved from the Ensembl website: https://ensembl.org/index.html) using the spliced alignment tool of Hisat2 (v2.7.10b) (21) with default parameters, and the information of read alignments was saved in BAM files for each sample. The BAM files were used to *de novo* reconstruct transcriptome using Stringtie (22). To identify the lncRNAs in PAMs, we merged the constructed transcriptome of each sample and compared it to the Ensembl reference transcriptome (Ensembl release 103, https://ftp.ensembl.org/pub/release-103/) to find novel transcripts using Gffcompare (23). The novel transcripts with a length size > 200 nt were subjected to coding-ability checks using CPC2 (24), CPAT (25), CNCI (26), and Pfam_scan (27). The non-coding sequences identified by all four types of the above software were considered lncRNAs, otherwise, they will be considered as mRNA. The expression levels of each transcript, including mRNAs and lncRNAs, were simultaneously normalized using the algorithm of Fragments Per Kilobase of Exon Model per Million mapped fragments (FPKM). The count matrix filled read number in each transcript was subjected to differential analysis using R package DEseq2 (v1.40.2) (28). The transcripts with criteria of |log2(fold-change)| > 1 (log2FC > 1) and *p* < 0.05 were considered as differentially expressed mRNA (DEMs) or lncRNA (DELs).

### 2.5 Construction of RNA subcellular localization classifier

The RNA subcellular localization classifier was designed using Pytroch and DGL packages (29). The framework of the classifier was constructed according to a previous study with appropriate modification, in which Li and colleagues trained a graph convolutional network (GCN) using small datasets of lncRNAs (30). In our study, training and test datasets were retrieved from the RNALocate v2.0 database (31), which recorded RNA information of experimental results of subcellular localization. We obtained a clean dataset containing 22270 RNA-associated subcellular localization entries after removing similar sequences and merging entries with redundant labels of subcellular localization. The RNA sequences were labeled with the “nucleus”, “cytoplasm”, and “nucleus_cytoplasm” (RNA located in both nucleus and cytoplasm). For the model, we added a two-head self-attention mechanism layer to extract high-level features after the GCN layer extracted features from sequences. The training process included 3 steps (1): RNA sequences were transformed to 5-mer sequences to construct de Bruijn graph; (2) 5-mer sequences were subjected to word embedding to generate node features of nodes in the graphs; (3) the constructed graphs were randomly classified into 10 parts to perform 10-fold cross-validation. The Accuracy (ACC), Macro Precision, Macro Recall, Macro F1-score, and area under the receiver operator characteristic (ROC) curve (AUC) were used as evaluation metrics to evaluate the performance of our model. The subcellular localization of mRNA and lncRNA were predicted using the voting results of the 10 models in 10-fold cross-validation, with 90% probability when one RNA sequence is predicted to locate in special subcellular localization. The source code and hyperparameters of the neural network models are listed in **Supplementary Table S1**.

### 2.6 MiRNA sequencing and quantification

The miRNA-seq libraries were constructed using VAHTS Small RNA Library Prep Kit for Illumina (Vazyme, Nanjing, China). Briefly, the secondary structure of the RNA was denatured at 70°C after mixing the RNA 3’ adapter with total RNA. The 5’ adapter was added using T4 RNA ligase at 25°C for 1 hour. Then, the first strand of cDNA was synthesized using reverse transcriptase with reverse transcription primers. The cDNA was amplified using the high-fidelity polymerase (Sangon Biotech, Shanghai, China). These libraries were checked using 6% high-resolution polyacrylamide gel electrophoresis (PAGE), and gels with the fragments at 140-160 bp were collected. The purified fragments from the collected gels were checked using Qubit (Life Technologies, Carlsbad, CA, USA) and Agilent 2100 Bioanalyzer (Agilent Technologies, CA, USA). All purified libraries were sequenced on an Illumina NovaSeq platform. The raw reads of miRNA-seq were filtered using Fastp (20). The adapter sequences, the reads containing no insert sequences, and reads with a Q20 ratio below 60% were removed, and only reads with length size ranging from 18~36 bp were kept. The clean reads were mapped to the miRbase and Rfam to identify known miRNAs. The raw read counts estimated by MirDeep2 (32) were used to analyze the differentially expressed miRNAs (DEmiRNAs) among different groups using DEseq2 (v1.34.0) (28). The miRNAs with the thresholds of |log2FC| >1 and *p* < 0.05 were considered DEmiRNAs.

### 2.7 Primer designing and cDNA synthesis

For lncRNAs and mRNAs, the specific primers used in RT-qPCR assay were designed using NCBI primer-blast software (https://www.ncbi.nlm.nih.gov/tools/primer-blast/), of which specificities were checked in the Refseq mRNA database of pigs (taxid: 9823). Total RNA was reversed transcribed to complementary DNA (cDNA) of lncRNAs and mRNAs using Master Premix for first-strand cDNA synthesis for Real-Time PCR (Foregene, Chengdu, China). For miRNA, the forward primers of miRNAs were designed based on the corresponding miRNA sequences (the U bases were replaced using T bases). Total RNA was reversed and transcribed to the cDNA of mRNAs using the Mir-X miRNA First-Strand Synthesis Kit (Takara, Dalian, China). The cDNA of lncRNA, mRNA, and miRNA was used as the template for downstream quantitative real-time PCR.

### 2.8 Quantitative real-time PCR (qPCR)

The qPCR was performed on a Bio-Rad C1000 Touch Thermal Cycler according to the manufacturer’s instructions. The qPCR reaction system was prepared using a SYBR II master mix kit (TAKARA, Dalian, China). A 10 μL qPCR reaction system was constructed using 0.5 μL forward primer (10 μM), 0.5 μL Reverse Primer (10 μM), 1 μL cDNA, 5 μL SYBR mix, and 3 μL ddH2O. The amplification reaction of the qPCR reaction system was executed under the following program: pre-denaturation at 94°C for 10 s, followed by 40 cycles of denaturation at 94°C for 5 s and annealing/extension at 58.8°C for 20 s. The melting curve analysis was performed from 65 to 95°C with an increment of 0.5°C. All the RT-qPCR Ct-values were normalized to that of the *ACTB* (for mRNA and lncRNA) or *U6* (for miRNA) and NCG group using the 2^−ΔΔCt^ method.

### 2.9 Functional annotation and pathway analysis

Gene Ontology (GO) enrichment and Kyoto Encyclopedia of Genes and Genomes (KEGG) pathway analysis were performed using the Database for Annotation, Visualization and Integrated Discovery (DAVID) (33). The enriched GO-BP terms or KEGG pathways with a *p* < 0.05 were considered significant.

### 2.10 Statistical analysis

Statistical analyses, including T-test and One-way ANOVA, were conducted on R software. The *p* < 0.05 was considered significant. The “*” represents “*p* < 0.05” in a statistical test, and the “**” represents “*p* < 0.01” in a statistical test.

## 3 Results

### 3.1 Genome-wide identification and characterization of lncRNAs in PAMs

**Figure 2:**
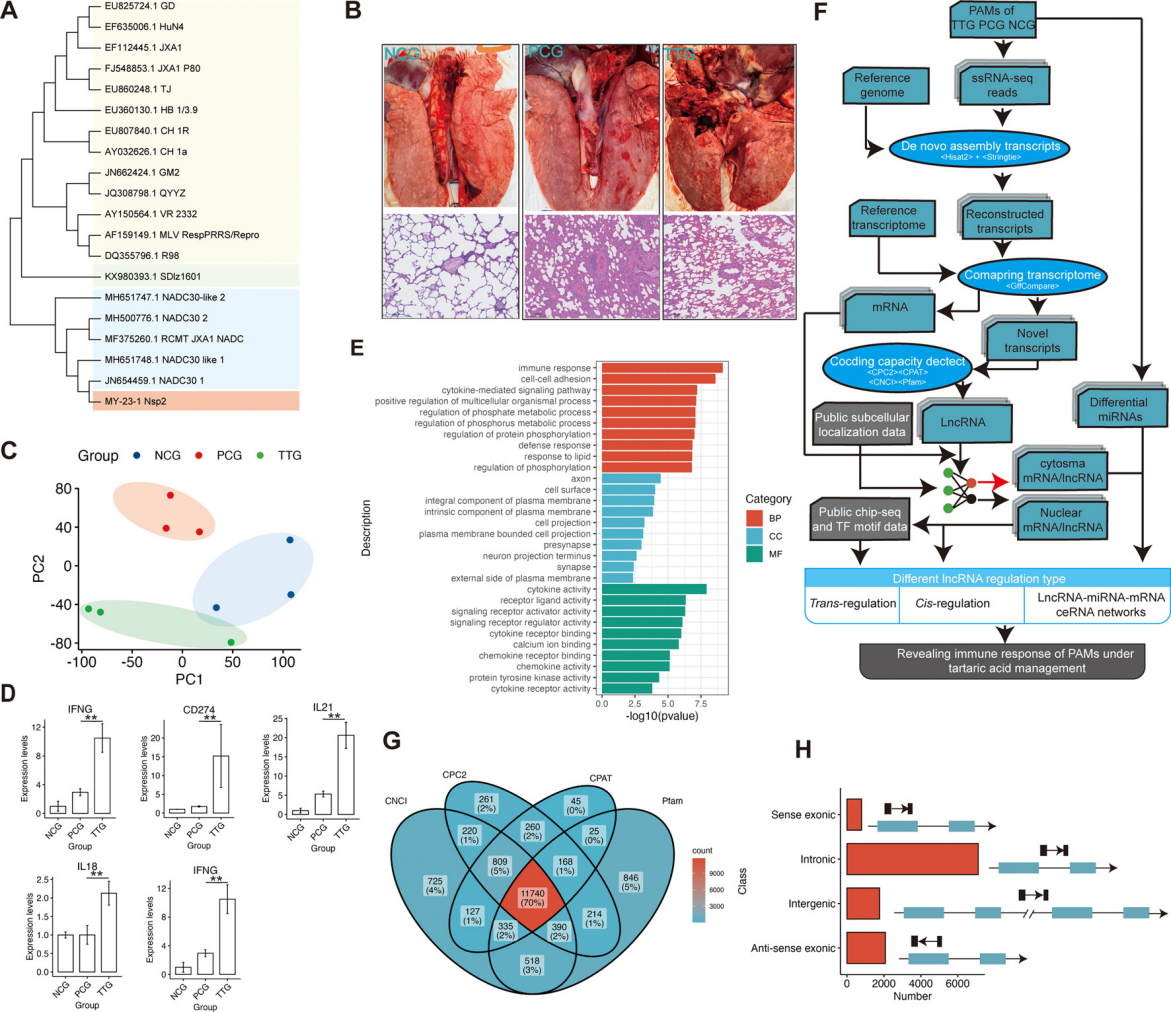
Histology analysis of lungs and *de novo* reconstruction of long non-coding RNAs of PAMs when PRRSV infection after administration of tylvalosin tartrate. **(A)** Phylogenetic analysis of MY-23-1 strain using *Nsp2* of PRRSV. **(B)** H&E analysis of pig lungs of the negative control group (NCG), the positive control group (PCG), and the Tylvalosin-treated group (TTG). The scale bars represent 100 μm length size. **(C)** Principal component analysis of transcriptome based on the gene expression levels. PC1 and PC2 represent the first principal component and second principal component, respectively. **(D)** Representative immune-related genes that significantly upregulated when PRRSV infection after administration of tylvalosin tartrate. The “**” represents “p < 0.01” in a statistical test of DEseq2. **(E)** Gene Ontology enrichment analysis of differentially expressed genes (DEGs) among NCG, PCG, and TTG. The BP, CC, and MF represent the category of biological processes, cellular components, and molecular functions, respectively. **(F)** Schematic diagram of the bioinformatics pipeline for the transcriptome assembly, identification and characterization of lncRNAs, and analysis of their *cis-* and *trans-*, and ceRNA regulatory roles. In brief, the clean reads were mapped to the reference genome using Hisat2, and the transcripts were constructed by Stringtie. LncRNAs were then predicted by CPC2, CNCI, CPAT, and Pfam-scan, and further, the subcellular localization of lncRNAs and mRNAs was simultaneously predicted using the deep-learning method. Finally, the immune functions and mechanisms of lncRNA and mRNA were analyzed based on subcellular localization. **(G)** Identification of lncRNAs using CPC2, CPAT, CNCI, and Pfam-scan. The transcripts that were simultaneously predicted as non-coding RNAs by the four types of algorithms were considered lncRNAs. **(H)** The classification of the lncRNAs.

In this study, the isolated PRRSV MY-23-1 strain was typed by sequencing *Nsp2*, *Orf5*, and *Orf7* genes of PRRSV. Phylogenetic analysis showed that MY-23-1 has undergone a significant amount of nucleotide variations compared to classical PRRSV strains, such as CH1a, GD, and GM2, while there were high degrees of sequence similarity of *Nsp2*, *Orf5*, and *Orf7* between MY-23-1 and the prevalent PRRSV strains in recent years, such as NADC30, NADC30-like, and the recombinant strain of NADC30 and JXA1 (**Figure 2A**, **Supplementary Figure S1A**). Pigs were classified into 3 groups, including NCG (*n* = 3, negative control), PCG (*n* =3, challenged with PRRSV MY-23-1), TTG (*n* = 3, treated with tylvalosin tartrate before PRRSV challenging). The animal experiment showed that lungs were seriously injured when pigs were challenged with PRRSV MY-23-1, while treatment of tylvalosin tartrate moderately relieved symptoms of lung injury (upper panel of **Figure 2B**). Histological examination showed that the piglets in PCG group presented obvious lung lesions, including disappearing lung structure and infiltrated necrotic debris, while treatment of tylvalosin tartrate (TTG) relieved these lesions (bottom panel of **Figure 2B**). We then isolated PAMs and performed ssRNA-seq to investigate the whole transcriptome response of PAMs. The ssRNA-seq showed that we obtained 59.46 M – 68.29 M raw reads among samples (**Supplementary Table S2**). The data of quality control showed that the Q30 read ratio and the clean read ratio ranged from 91.40% to 96.20% and 98.40% to 99.20%, respectively (**Supplementary Table S2**). The PCA analysis based on the gene-level quantification showed that the samples in TTG, PCG, and NCG were classified into corresponding groups, respectively (**Figure 2C**). Differential analysis of protein-coding genes showed that a total of 985 and 772 genes were significantly upregulated [*p* < 0.05 and log2(FC) > 1] and downregulated [*p* < 0.05 and log2(FC) < -1] in PAMs when pigs were challenged by PRRSV (PCG *vs.* NCG, **Supplementary Table S3**). The functional analysis found that these differentially expressed genes (DEGs) mainly enriched in the functions related to transmembrane signaling transduction and cytokine, such as the top 3 significantly enriched GO terms of transmembrane receptor protein tyrosine kinase activity, transmembrane receptor protein kinase activity, and cytokine activity (**Supplementary Figure S1B**). Analysis of the difference between TTG and PCG found that 786 and 188 genes were significantly upregulated and downregulated in PAMs when pigs were treated with tylvalosin tartrat (**Supplementary Table S3**). Some immune factors, such as *IFNG*, *CD274*, *IL21*, *IL18*, and *IRF1*, were found to maintain or slightly upregulate expression in PCG *vs.* NCG but significantly upregulated in TTG *vs.* PCG (**Figure 2D**). Functional analysis showed that these DEGs were mainly enriched in the immune activity of PAMs, such as the significantly enriched GO terms of the immune response, cell-cell adhesion, and defense response (**Figure 2E**). These data suggested that tylvalosin tartrate reduced lung injury caused by PRRSV infection through immune regulation of PAMs at the transcriptional level.

Mapping reads to the reference genome found that the major reads (68.03% - 74.90%) of ssRNA-seq were allocated into intergenic, intronic, and UTR regions, and only 25.10% - 31.96% reads were allocated into known protein-coding regions, indicating pig PAMs were a type of cell with a high diversity of transcript types (**Supplementary Figure S1C**). To obtain a comprehensive profile of lncRNAs of PAMs, we performed *de novo* transcriptome assembly for our ssRNA-seq data (**Figure 2F**). In total, we constructed 95607 transcripts, and a stringent pipeline was designed to identify 11740 high-confidence lncRNAs from the *de novo* constructed transcriptome after filtering by four types of coding ability predictors, including CPC2, CNCI, CPAT, and Pfam (**Figures 2F, G**). According to genomic location, these lncRNAs were further classified into four types: 791 sense-exonic lncRNAs, 7107 intronic lncRNAs, 1765 intergenic lncRNAs, and 2077 anti-sense exonic lncRNAs (**Figure 2H**). Compared to protein-coding RNAs, the lncRNAs showed higher average expression levels (**Supplementary Figure S1D**) and lower expression complexity (**Supplementary Figure S1E**).

### 3.2 Attention mechanism-improved graph convolutional network classified PAM transcripts into nucleus and cytoplasm

**Figure 3:**
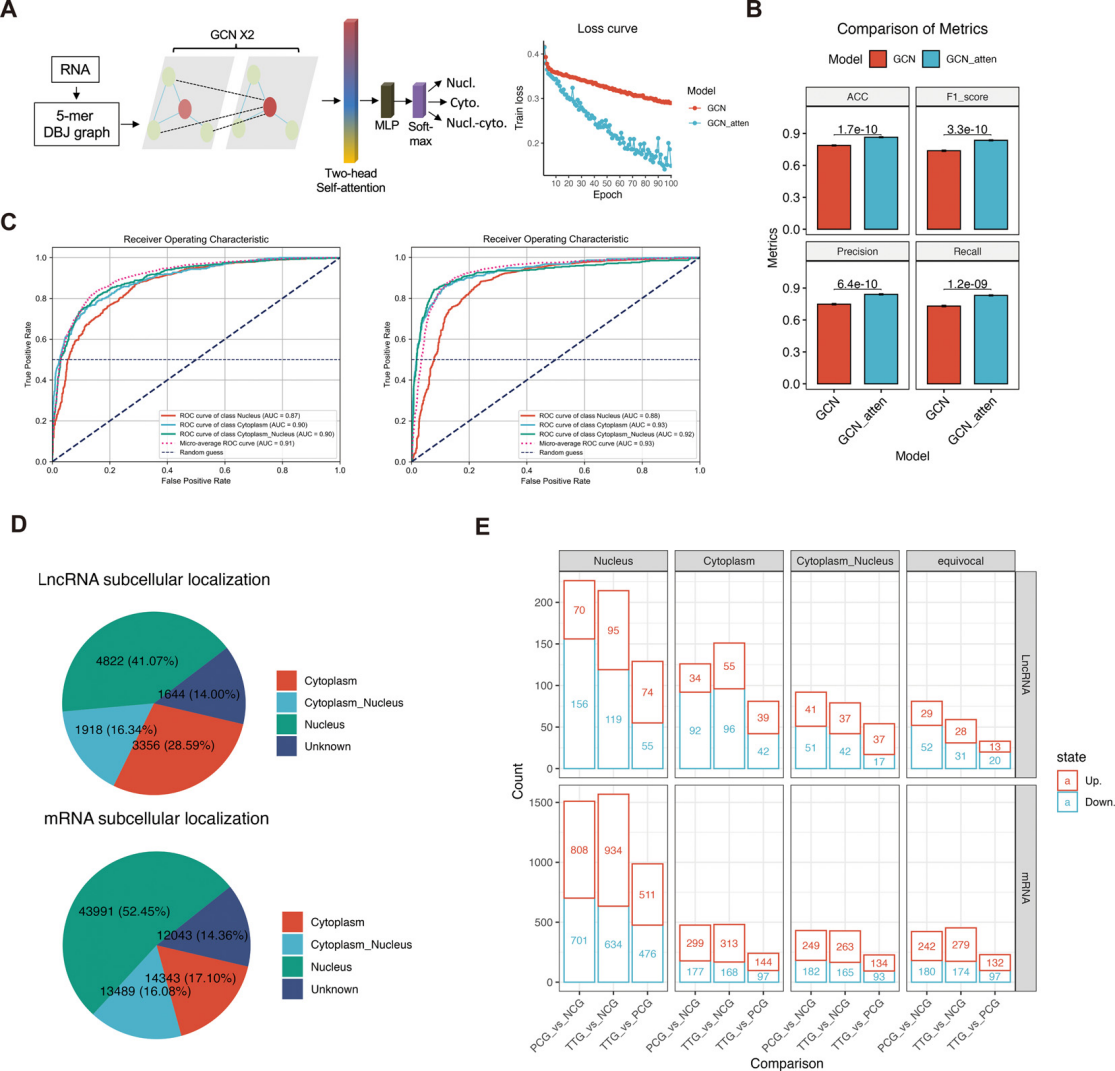
Attention mechanism-improved graph convolutional network (GCN_atten) predicted the subcellular localization of lncRNA and mRNAs. **(A)** Sequence transformation and architecture of the neural network (left panel). The values of loss function during epoch training (right panel). **(B)** Comparisons of the accuracy, F1-score, precision, recall, and ROC of prediction between RNALocate and GCN_atten in 10-fold cross-validation. **(C)** Comparison of receiver operating characteristic curves between RNALocate and GCN-atten in different classification targets (cytoplasm, nucleus, and cytoplasm_nucleus). **(D)** Prediction of subcellular localization of lncRNA and mRNAs using GCN_atten. **(E)** Differential analysis of lncRNA and mRNA based on their subcellular localization.

The mechanism of lncRNA is complex and diverse, and the subcellular localization of lncRNAs is crucial for understanding lncRNA functions. Currently, some computational methods have been proposed to predict lncRNA subcellular localization based on the known experimental data. To the best of our knowledge, the best predictor is GraphLncLoc (30), which predicts lncRNA subcellular localization using graph convolutional networks (GCN) based on sequence-to-graph transformation. However, the performance of the GraphLncLoc was limited (accuracy = 0.579) in the cases of less training lncRNA sequences and multi-class classification (769 lncRNAs for four different subcellular localizations). Recent studies have revealed attention mechanism is useful in sequence analysis (34). Therefore, we prepared a GCN_atten, an attention mechanism-improved GCN, to predict both lncRNA and mRNA subcellular localization (**Figure 3A**). This model mainly contained two graph convolutional layers, one two-head self-attention layer, one multilayer perceptron layer, and a soft-max layer. A total of 20672 mRNA and 1598 lncRNA sequences that had been experimentally marked with subcellular localization were retrieved from the RNALocate v2.0 database (31) and then were converted to 5-mer De Bruijn graphs to train our model. Our data showed that the values of the loss function of GCN_atten converge faster than a previous existing GCN model (GraphLncLoc) during the epoch training (**Figure 3A**). The GCN_atten has excellent performance in terms of key evaluation metrics. The accuracy, F1-score, precision, and recall of prediction achieved 0.865%, 0.836, 0.841, and 0.831, and were all significantly higher than that of GraphLncLoc (accuracy = 0.788, F1-score = 0.738, precision = 0.748, recall = 0.731) in 10-fold cross-validation, respectively (**Figure 3B**). On the other hand, the ROC curves of GCN_atten achieved 0.88%, 0.93%, and 0.92% for the classification of RNA in the nucleus, cytoplasm, and both the nucleus and cytoplasm, which were higher than that of GCN (**Figure 3C**). Our data indicate that we obtained a robust RNA subcellular localization classifier. Therefore, this classifier can be used for downstream analysis.

Analysis of lncRNAs of PAMs showed that 4822 (41.07%), 3356 (28.59%), 1918 (16.34%), and 1644 (14.00%) lncRNAs were predicted to be located in the nucleus, cytoplasm, both nucleus and cytoplasm and unknown subcellular localization, respectively (**Figure 3D**). On the other hand, we also predicted the mRNA subcellular localization, a total of 43991 (52.45%), 14343 (17.10%), 13489 (16.08%), and 12043 (14.36%) mRNAs were predicted to be located in the nucleus, cytoplasm, both nucleus and cytoplasm and unknown subcellular localization, respectively (**Figure 3D**). Comparing different experimental groups showed that 569, 358, 225, and 173 differentially expressed lncRNAs (DELs) were found in the nucleus, cytoplasm, both nucleus and cytoplasm, and unknown subcellular localization, respectively. Moreover, a total of 4064, 1198, 1086, and 1104 differentially expressed mRNAs (DEMs) were found in the nucleus, cytoplasm, both nucleus and cytoplasm, and unknown subcellular localization, respectively (**Figure 3E**, **Supplementary Table S4**). Overall, our deep-learning model predicted the subcellular localization of mRNAs and lncRNAs, which facilitated an opportunity further to investigate different regulatory mechanisms of lncRNA in PAM cells.

### 3.3 Screening miRNAs response to PRRSV and tylvalosin tartrate

**Figure 4:**
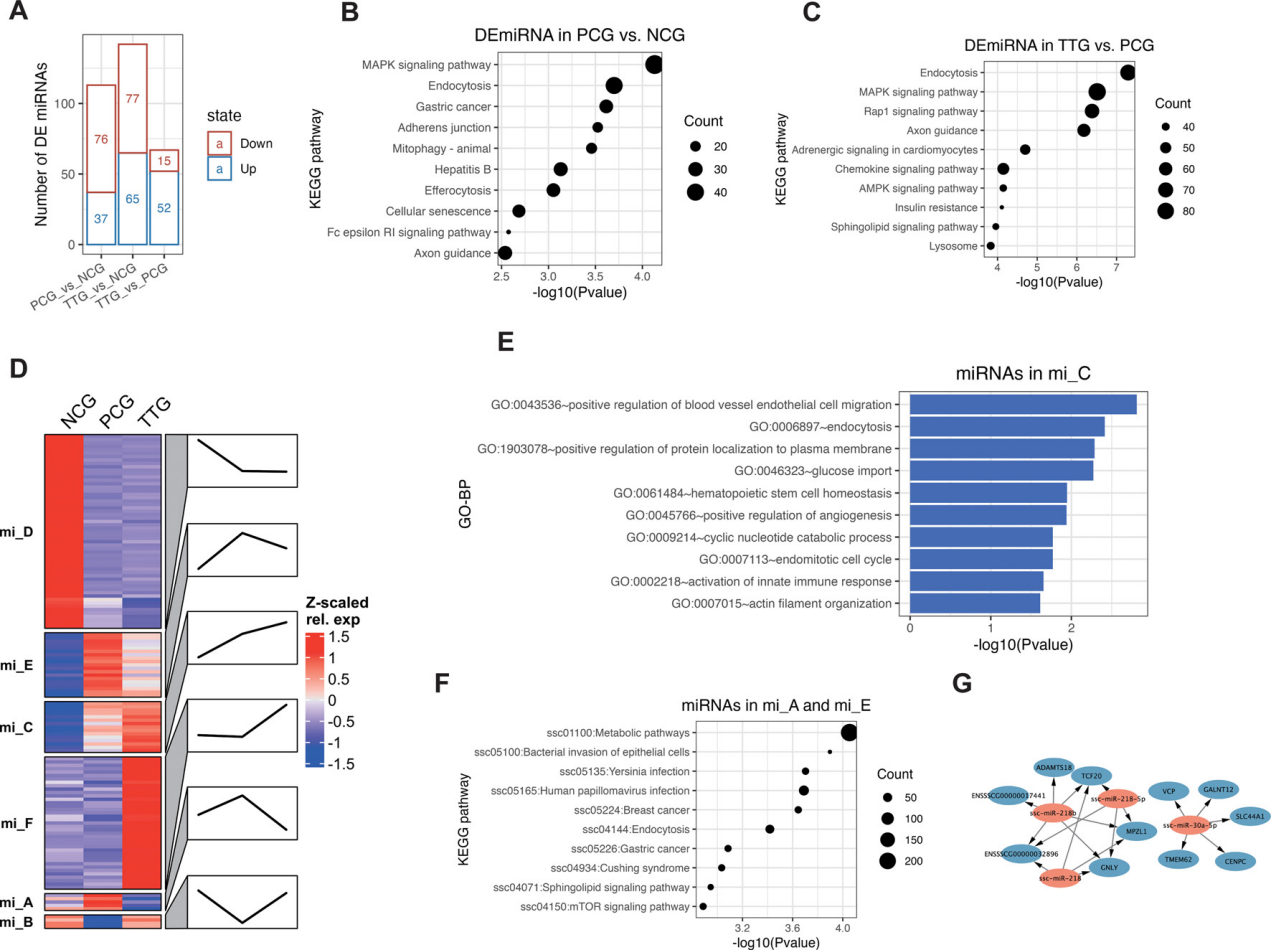
MiRNA changes in PAMs when PRRSV infection after administration of tylvalosin tartrate. **(A)** Differentially expressed miRNAs (DEmiRNAs) among NCG, PCG, and TTG. **(B)** Kyoto Encyclopedia of Genes and Genomes (KEGG) pathway analysis of DEmiRNAs between PCG and NCG. **(C)** KEGG pathway analysis of DEmiRNA between TTG and PCG. **(D)** K-means clustering of DEmiRNAs. Line plots annotated the change trends of expression levels of the corresponding group. **(E)** GO analysis of tylvalosin tartrate-enhanced favorable miRNAs. **(F)** GO analysis of tylvalosin tartrate-reversible harmful miRNAs. **(G)** Regulatory networks of tylvalosin tartrate-reversible favorable miRNAs.

LncRNAs often interact with miRNAs to function as ceRNAs. In this study, we further performed miRNA-seq to detect miRNA changes. MiRNA-seq showed that we obtained 10.49 – 13.58 M raw reads among samples (**Supplementary Table S2**). Differential analysis of miRNAs showed that 76, 77, and 15 miRNAs were significantly upregulated [*p* < 0.05 and log2(FC) > 1, **Supplementary Table S5**] in the comparisons of PCG *vs.* NCG, TTG *vs.* NCG, and TTG *vs.* PCG, respectively. On the other hand, 37, 65, and 52 miRNAs were significantly downregulated [*p* < 0.05 and log2(FC) < -1, **Supplementary Table S5**] in the comparisons of PCG *vs.* NCG, TTG *vs.* NCG, and TTG *vs.* PCG, respectively (**Figure 4A**). KEGG pathway analysis of target genes found that the MAPK signaling pathway, endocytosis, and gastric cancer were the top 3 significantly enriched KEGG signaling pathways by the differentially expressed miRNAs (DEmiRNAs) of PCG *vs.* NCG (**Figure 4B**). The Endocytosis, MAPK signaling pathway and Rap1 signaling pathway were the top 3 significantly enriched KEGG signaling pathways by the DEmiRNAs of TTG *vs.* PCG (**Figure 4C**).

To further understand the miRNA’s response to PRRSV and tylvalosin tartrate, we performed a k-means clustering analysis for the DEmiRNAs. K-means clustering classified all DEmiRNAs into 6 groups (from mi_A to mi_F) with different expression patterns among NCG, PCG, and TTG (**Figure 4D**). The miRNAs in the mi_D were highly expressed in the NCG but lowly expressed in both PCG and TTG, representing the miRNAs that only responded to PRRSV challenging but not to tylvalosin tartrate. The miRNAs in the mi_F were lowly expressed in both NCG and PCG but upregulated in TTG, representing the miRNAs that only responded to tylvalosin tartrate but not to PRRSV challenging. The miRNAs in the mi_C were upregulated from both NGC to PCG and PCG to TTG, representing the miRNAs that responded to PRRSV challenging and tylvalosin tartrate promotes this response; we refer to these miRNAs as potential tylvalosin tartrate-enhanced favorable miRNAs (TEFMi). Functional analysis of these TEFMi found that their target genes were significantly enriched in angiogenesis and innate immune-related functions, such as the top enriched GO-BP terms of positive regulation of blood vessel endothelial cell migration, positive regulation of angiogenesis, and activation of innate immune response (**Figure 4E**). The miRNAs in the mi_A, mi_B, and mi_E were only highly or lowly expressed in PCG, representing the miRNAs that responded to PRRSV challenging, and tylvalosin tartrate reversed this response. Among the mi_A, mi_B, and mi_E, the miRNAs in mi_A and mi_E were only highly expressed in PCG; we refer to these miRNAs as potential tylvalosin tartrate-reversible harmful miRNAs (TRHMi). KEGG pathway analysis of the target genes of these TRHMi found that they were significantly enriched in the infection-related signaling pathways, such as the bacterial invasion of epithelial cells, yersinia infection, and human papillomavirus infection (**Figure 4F**). On the other hand, the miRNAs in mi_B were only lowly expressed in PCG; we refer to these miRNAs as potential tylvalosin tartrate-reversible favorable miRNAs (TRFMi). Three of the four miRNAs in mi_B, including *ssc-miR-30a-5p* (35), *ssc-miR-218-5p* (36), and *ssc-miR-218* (37) were previously reported to repress inflammation in macrophages, respiratory organ, and nephropathy, respectively. As shown in **Figure 4G**, miRNAs in mi_B were predicted to target 11 mRNAs, including *GNLY*, an inflammatory gene (**Figure 4G**).

### 3.4 Construction of lncRNA-miRNA-mRNA ceRNA networks based on cyto mRNA and lncRNAs

**Figure 5:**
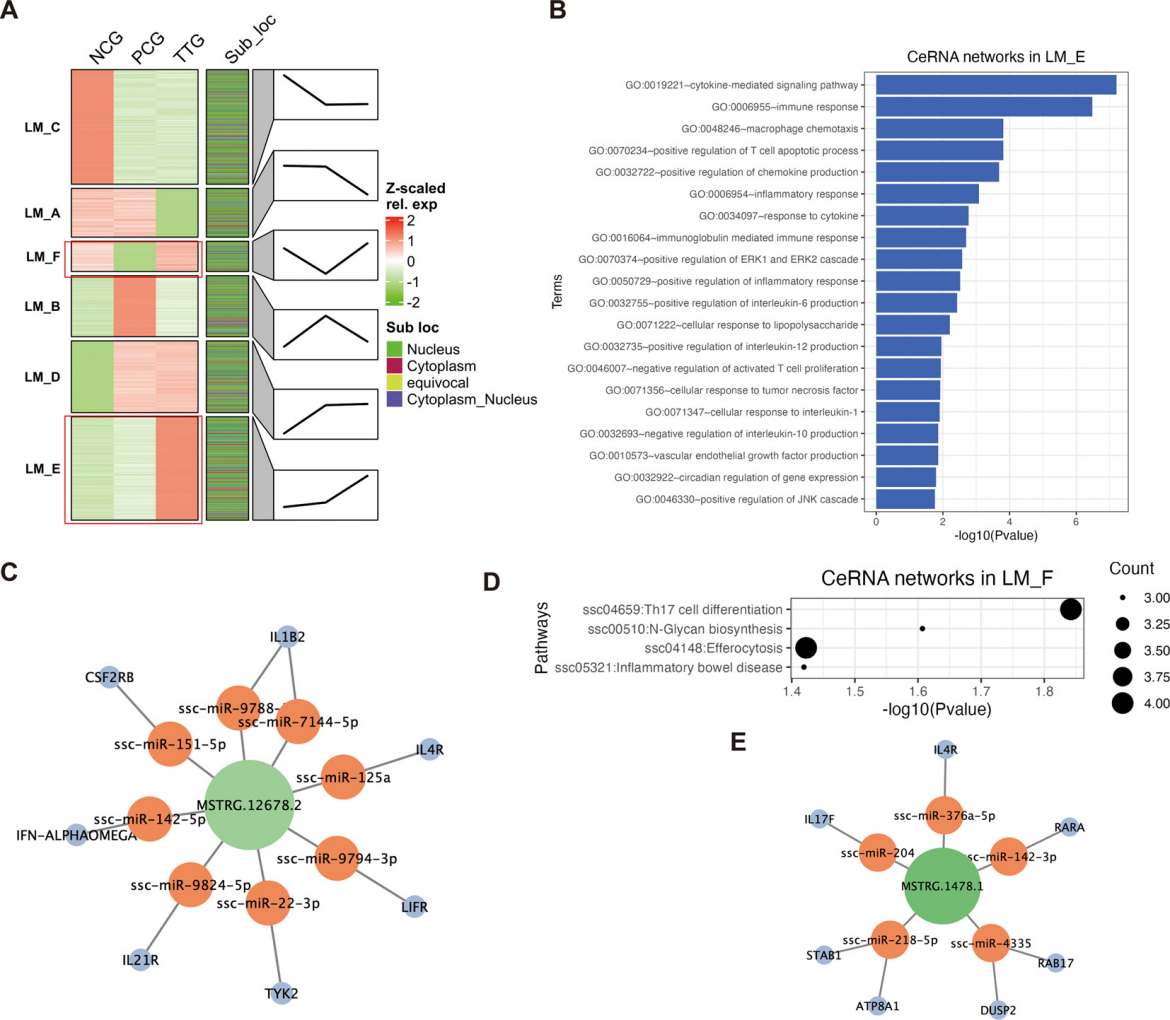
Construction of ceRNA networks based on the cytoplasmic lncRNAs and mRNAs. **(A)** K-means clustering of DELs and DEMs. Each transcript was marked by its subcellular localization. Each cluster (LM_A, LM_B, LM_C, LM_D, LM_E, and LM_F) represents a co-expression group. Line plots annotated the change trends of expression levels of the corresponding group. **(B)** GO enrichment of ceRNA networks in LM_E. **(C)** Key lncRNA *MSTRG.12678.2* regulated ceRNA networks LM_E. **(D)** KEGG pathway analysis of ceRNA networks in LM_F. **(E)** Key lncRNA *MSTRG.1478.1* regulated ceRNA networks in LM_F.

To understand the lncRNA response to PRRSV and tylvalosin tartrate, we performed a k-means clustering analysis for both DELs and DEMs to detect their co-expression patterns. K-means clustering classified all DELs and DEMs into 6 co-expression groups (from LM_A - LM_F) with different expression patterns (**Figure 5A**). Among these co-expression groups, the lncRNAs in the LM_E were lowly expressed in the NCG but upregulated from both NGC to PCG and PCG to TTG, representing the lncRNAs that positively responded to PRRSV challenging and tylvalosin tartrate promoted this response, we refer to these lncRNAs as potential tylvalosin tartrate-enhanced favorable lncRNAs (TEFLs). On the other hand, the lncRNAs in LM_F were only lowly expressed in PCG; we refer to these lncRNAs as potential tylvalosin tartrate-reversible favorable lncRNAs (TRFLs) (**Figure 5A**).

LncRNA and mRNA binding to shared miRNA to form lncRNA-miRNA-mRNA ceRNA network is an important regulatory mechanism in the post-transcriptional process. Based on our miRNA data, we further extracted cytoplasmic lncRNAs and mRNAs from each co-expression group to construct lncRNA-miRNA-mRNA ceRNA networks for the TEFLs and TRFLs. For the cytoplasmic TEFLs, 93 lncRNAs were found to regulate 456 mRNA via interacting with 347 miRNAs (**Supplementary Table S6**). The cytoplasmic TEFLs were significantly enriched in immune response-related GO terms via corresponding ceRNA networks. For instance, the top 5 enriched GO terms were cytokine-mediated signaling pathway, immune response, macrophage chemotaxis, positive regulation of T cell apoptotic process, and positive regulation of chemokine production (**Figure 5B**). The most highly expressed TEFL in the ceRNA networks was *MSTRG.12678.2*, which was found to regulate 7 key immune factors (*IL1B2*, *IL4R*, *LIFR*, *TYK2*, *IL21R*, *IFN*-*ALPHAOMEGA*, and *CSF2RB*) via interacting with 8 miRNAs (*ssc-miR-9788*, *ssc-miR-7144-5p*, *ssc-miR-125a*, *ssc-miR-9794-3p*, *ssc-miR-22-3p*, *ssc-miR-9824-5p*, *ssc-miR-142-5p*, and *ssc-miR-151-5p*) (**Figure 5C**). For the cytoplasmic TRFLs, 42 lncRNAs were found to regulate 107 mRNAs via interacting with 346 miRNAs (**Supplementary Table S7**). The cytoplasmic TRFLs were also found to be significantly enriched in immune response-related GO terms via corresponding ceRNA networks, such as the top enriched GO-BP terms of the cytokine-mediated signaling pathway, regulation of immune response, and positive regulation of T cell differentiation (**Supplementary Figure S2**). KEGG pathway analysis of cytoplasmic TRFLs found that they were significantly enriched in four signaling pathways, including Th17 cell differentiation, N-Glycan biosynthesis, efferocytosis, and inflammatory bowel disease (**Figure 5D**). The most highly expressed TRFL in the ceRNA networks was *MSTRG.1478.1*, which was found to regulate 7 key Th17 cell differentiation and efferocytosis-related mRNAs (*IL4R*, *RARA*, *RAB17*, *DUSP2*, *ATP8A1*, *STAB1*, *IL17F*) via interacting with 5 miRNAs (*ssc-miR-376a-5p*, *ssc-miR-142-3p*, *ssc-miR-4335*, *ssc-miR-218-5p*, and *ssc-miR-204*) (**Figure 5E**).

### 3.5 LncRNA plays *cis-* and *trans-*regulatory roles in the nucleus of PAMs

**Figure 6:**
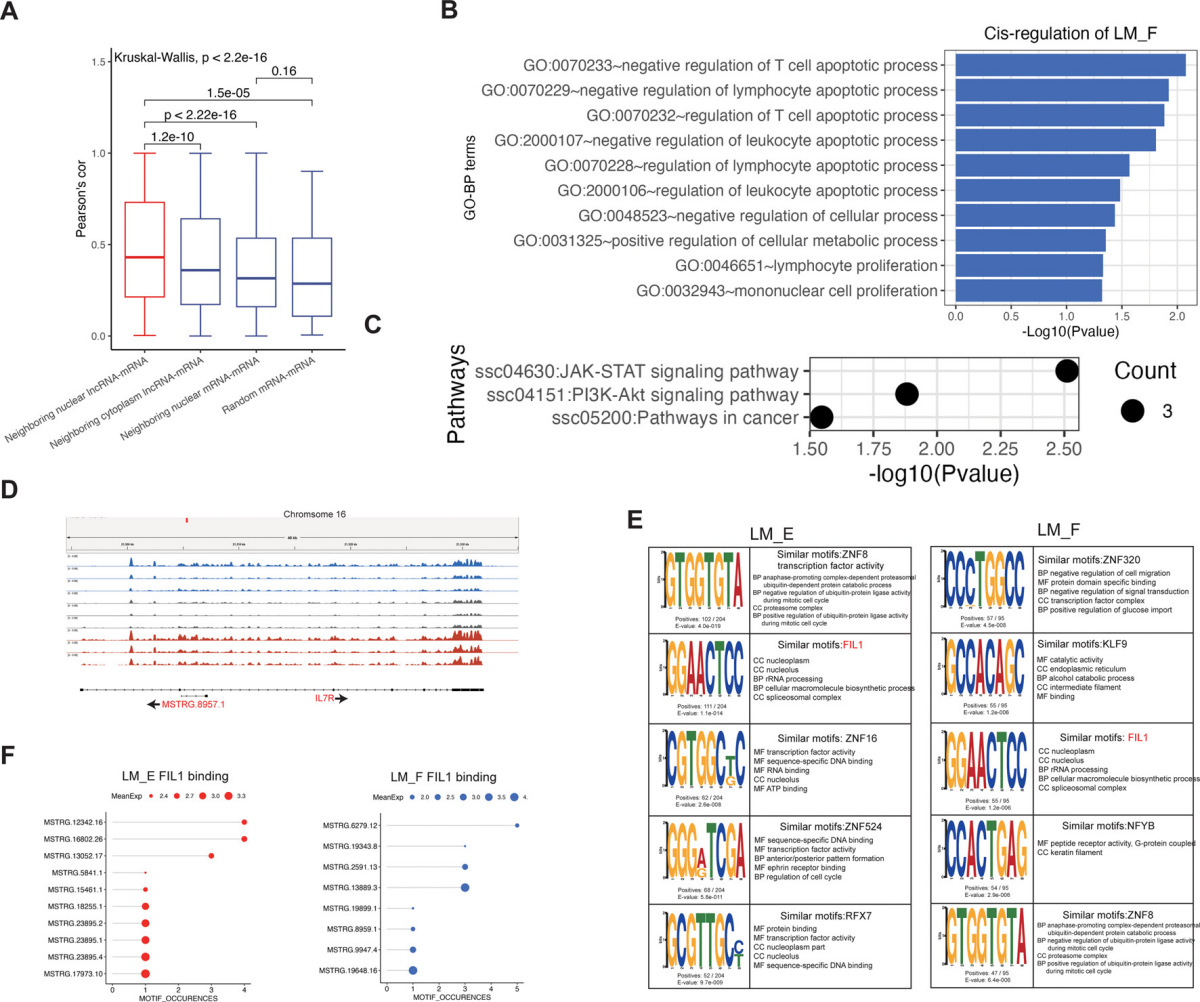
Cis- and trans-regulation of nucleus lncRNAs. **(A)** Comparison of the co-expressed correlations of neighboring nuclear lncRNA-mRNA, neighboring cytoplasmic lncRNA-mRNA, neighboring nuclear mRNA-mRNA, and random mRNA-mRNA groups. The significant differences between these groups were calculated using the Kruskal-Wallis test. **(B, C)** GO enrichment and KEGG pathway analysis of *cis-*regulation of lncRNAs in LM_F. **(D)** Anti-sense intronic lncRNA *MSTRG.8957.1* was found to potentially regulate IL7R in a *cis-*regulatory way. **(E)** Top 5 significantly enriched motifs in the lncRNA sequences in the LM_F and LM_E. **(F)** Screening key lncRNAs binding to FIL1.

In this study, the major lncRNAs were classified into the nucleus of subcellular localization, suggesting lncRNAs play important roles on the transcription level. To investigate the *cis-*regulation of nucleus lncRNAs, we explored the relationship of these lncRNAs and their neighboring protein-coding genes. Co-expression analysis on the neighboring nucleus lncRNA-mRNA pairs showed that many of them have a positive correlation. To determine the molecular mechanism of *cis-*regulation, we selected three other types of pairs as groups for comparison, including neighboring cytoplasmic lncRNA-mRNA pairs, neighboring nucleus mRNA-mRNA pairs, and random nucleus mRNA-mRNA pairs. These comparison results showed that the difference in co-expression of two types of mRNA-mRNA pairs is not statistically significant (p = 0.16). However, neighboring nucleus lncRNA-mRNA pairs have the significantly highest co-expression relationship than other pairs (t-test, *p* < 0.01, **Figure 6A**). These findings suggest that nuclear lncRNAs have a significantly higher regulation by positively *cis-*regulating their neighboring gene expression than cytoplasmic lncRNAs.

For the nuclear TEFLs (nuclear lncRNAs in LM_E) in the co-expression groups, 25 lncRNAs were found to potentially regulate their 19 neighboring genes in a *cis-*regulation way. These genes were found to be significantly involved in more varied biological processes, of which GO-BP terms were involved in macromolecule biosynthetic process, immune, and growth (**Supplementary Figure S3**). On the other hand, we found 11 nuclear TRFLs (nuclear lncRNAs in LM_F) potentially regulated their 9 neighboring genes (*FKBP5*, *USP6NL*, *SSH2*, *IL7R*, *CCND3*, *MLXIP*, *ANKRD33B*, *AKT2*, and *ENSSSCG00000030241*) in a *cis-*regulation way. GO enrichment showed that these genes were involved in lymphocyte apoptotic process-related biological processes, such as the top 3 significantly enriched GO-BP terms of negative regulation of T-cell apoptotic process, negative regulation of lymphocyte apoptotic process, and regulation of T-cell apoptotic process (**Figure 6B**). KEGG pathway analysis of these genes showed that they were significantly enriched in 3 signaling pathways, including the JAK-STAT signaling pathway, PI3K-Akt signaling pathway, and pathways in cancer (**Figure 6C**). The most highly expressed nuclear TRFL was *MSTRG.8957.1*, an anti-sense intronic lncRNA, which was found to potentially regulate *IL7R*, an important regulator for both humoral and cellular immunity (38), in a *cis-*regulatory way (**Figure 6D**).

To predict the *trans-*regulation of lncRNAs, we performed motif enrichment based on the RNA sequences of nuclear TEFLs and TRFLs. Of the motifs identified, “GTGGTGTA”, “GGAACTCC”, “CGTGGCKC”, “GGGRTCGA”, and “GCGTTGCY” were the most dominant motifs for sequences of the TEFLs. These five motifs were found to be similar to known binding motifs of ZNF8, FIL1, ZNF16, ZNF524, and RFX7, respectively. Gene ontology for motifs (GOMO) analysis (39) showed that these motifs were involved in proteasomal ubiquitin-dependent protein catabolic processes, nuclear processes, and transcription factor (TF) activity (**Figure 6E**). On the other hand, “CCSTGGCC”, “GCCACAGC”, “GGAACTCC”, “CCACTGAG”, and “GTGGTGTA” were the most dominant motifs for the sequences of TRFLs. These five motifs were found to be similar to known binding motifs of ZNF320, KLF9, FIL1, NFYB, and ZNF8, respectively. GOMO analysis suggests that these motifs were involved in negative regulation of cell migration, catalytic activity, and nuclear processes (**Figure 6E**). Interestingly, an emerging important anti-inflammation cytokine, IL37 (aliases FIL1) (40), was found to be enriched as dominant similar motifs for the sequences of both TEFLs and TRFLs, suggesting its important role during the administration of tylvalosin tartrate in our study. Based on the motif occurrences of IL37 on the RNA sequences and expression levels, six highly expressed lncRNAs with more than 3 IF37 binding sites were selected as anti-inflammation lncRNA candidates in a *trans-*regulation way, including three TEFLs (*MSTRG*.*12342*.*16*, *MSTRG*.*16802*.*26*, and *MSTRG*.*13052*.*17*) and three TRFLs (*MSTRG*.*6279*.*12*, *MSTRG*.*2591*.*13*, and *MSTRG*.*13889*.*3*) (**Figure 6F**).

### 3.6 Validation of whole transcriptome using RT-qPCR

**Figure 7:**
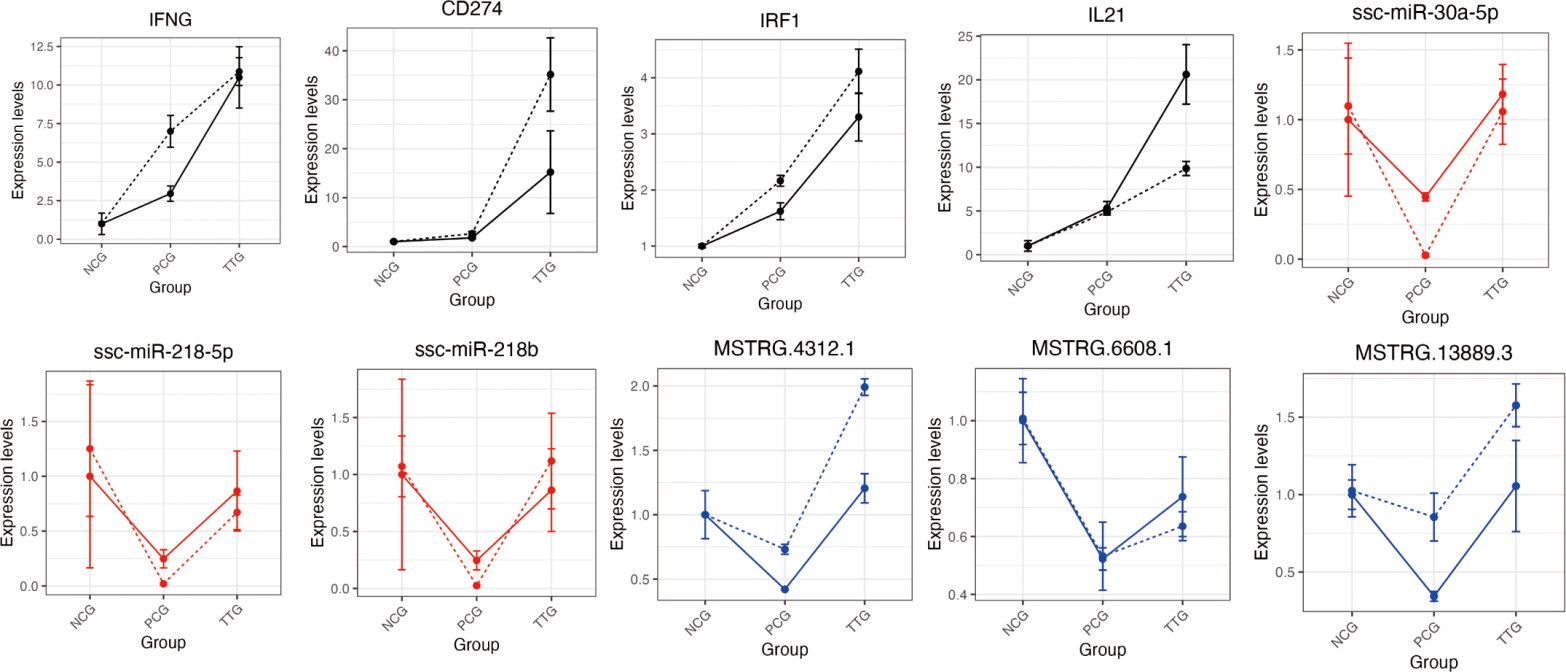
Validation of 10 differentially expressed mRNAs, miRNAs, and lncRNAs using RT-qPCR. The black, blue, and red lines represent the mRNAs, lncRNAs, and miRNAs, respectively. The solid and dashed lines represent the results of ssRNA-seq/miRNA-seq and RT-qPCR, respectively. The expression was normalized to the *ACTB* (for mRNAs and lncRNAs) or *U6* (for miRNAs) gene and NCG group. The data shows the means and standard error of three independent experiments. Two technical replicates were set for one individual experimental replicate in RT-qPCR.

To validate the ssRNA-seq and miRNA-seq results, we selected four tylvalosin tartrate enhanced immune factors (*IFNG*, *CD274*, *IRF1*, and *IL21*), three TRFMis (*ssc-miR-30a-5p*, *ssc-miR-218-5p*, and *ssc-miR-218b*), and three TRFLs (*MSTRG.6608.1*, *MSTRG.13889.3*, and *MSTRG.4312.1*) and evaluated their expression levels using RT-qPCR (Primers are listed in **Supplementary Table S8**). Regardless of differences in the magnitude of fold-changes, expression of all these selected mRNAs determined by RT-qPCR displayed changes in the same direction as that observed using ssRNA-seq or miRNA-seq (**Figure 7**). Therefore, expression levels, as demonstrated in this study by sequencing, are reliable.

## 4 Discussion

PRRS is one of the most economically devastating diseases affecting the swine industry worldwide (41). Some studies suggest that host genetics play a role in susceptibility to respiratory diseases, including PRRS (42). Because PAMs are the major target cells of PRRSV, previous transcriptome studies have identified different types of genetic factors based on the comparisons between normal PAMs and PRRSV-infecting PAMs *in vitro* and *in vivo* (18, 43, 44). Considering the complexity and relative disorder of host gene expression in disease states, it is difficult to directly identify favorable and harmful genes of PAM during PRRSV infection. In this study, an *in vivo* model was set to evaluate the anti-PRRSV effect of tylvalosin tartrates. Our data showed that the administration of tylvalosin tartrates relieved symptoms of lung injury, which was in line with a previous animal experiment (16). Using ssRNA-seq and miRNA-seq, we first obtained the landscapes of the whole transcriptome among healthy, PRRSV-infected, and PRRS-relieved piglets. Our data showed that tylvalosin tartrates increased the expression of many cytokine-related genes, such as *IFNG*, *IL18*, and *IL21*, suggesting that the stimulation of tylvalosin tartrates promotes the anti-inflammatory function of PAM cells during PRRSV infection.

Host lncRNAs were previously reported to regulate innate immune response to an RNA virus (45). In this study, we designed a stringent pipeline for lncRNA identification and found > 10 K high-confidence lncRNAs, this number was similar to spleens (46) but much higher than that of other non-immune tissues, such as fat (47), skeletal muscle (48), and ovary (49), which suggests that lncRNA might be an important type of ncRNA involved in immune regulation of PAMs. On the other hand, our data showed that intronic lncRNAs account for the majority of all PAM lncRNAs, which might suggest that PAM kept an active alternative splicing process during PRRSV infection. It has been reported that the subcellular localization of lncRNAs is different, and the mechanisms of lncRNA subcellular localization are diverse (10). The multiple subcellular localization of lncRNAs is very common (For example, *H19*) (50, 51). Although some computational methods have been proposed to predict lncRNA subcellular localization, few classifiers considered the multiple subcellular localization of lncRNAs. In this study, the neural network classifier we designed not only improves on the training data and model but also introduces multiple classifications in terms of classification types and our model has excellent performance in terms of key evaluation metrics. Based on this model, we predicted the subcellular localization of lncRNA and mRNA, which will contribute to the subsequent analysis of regulatory mechanisms of PAMs. MiRNAs play important roles in the communication among different genetic components and the regulation of disease development (52). Recent studies have emphasized that miRNAs mainly act as competing endogenous RNAs to regulate cell functions in the cytoplasm. To comprehensively screen key miRNAs response to PRRSV infection and tylalosin tartrate treatment, we performed miRNA-seq in our PAM samples. Endocytosis is an important mechanism for the virus infecting hosts, which commonly occurs in the process of virus invasion mediated by the host’s channel proteins, such as in the infection processes of white spot syndrome virus and influenza virus (53, 54). In this study, the pathway of endocytosis was significantly enriched by the DEmiRNAs between TTG and PCG, which indicated that tylalosin tartrate treatment might mediate the infection process of PRRSV in PAMs and thus reduce the injury of the lungs. Previous studies have revealed the activation of MAPK signaling pathway is crucial for PRRSV infection (55, 56). Our data showed that the DEmiRNAs between TTG and PCG were significantly enriched in the MAPK signaling pathway, suggesting that tylalosin tartrate treatment might regulate PRRSV infection by miRNA-mediated gene expression. On the other hand, the identification of some previous studies confirmed marker miRNAs (*ssc-miR-30a-5p*, *ssc-miR-218-5p*, and *ssc-miR-218*) of PRRSV infection and inflammation gene (*GNLY*) indicated that we obtained robust results in the animal experiment and miRNA-seq.

The interaction between lncRNA and miRNA is the classic mode of action of lncRNA (11). In this study, we constructed lncRNA-related ceRNA networks based on cytoplasmic DELs. Our data showed that cytoplasmic TELs were widely involved in the immune regulation of PAM. Macrophage migration is the primary condition for exerting immune activity at the site of infection and injury, and macrophage chemotaxis reflects its migration ability, which is one of the main indicators for evaluating macrophage immune function (57). In this study, macrophage chemotaxis was one of the top 3 significantly enriched GO terms of cytoplasmic TEL-related ceRNA networks, which indicates that these lncRNAs play a crucial role in the migration of macrophages to lungs damaged by PRRSV. The clearance of apoptotic cells is essential for the maintenance of tissue homeostasis (58). In this study, efferocytosis was a significantly enriched KEGG pathway by the TRL-related ceRNA networks, which indicates that PRRSV damages efferocytosis-related functions when attacking PAMs, while tylvalosin tartrates protect PAMs via ceRNA networks by reversing the expression of related lncRNAs. In addition, we identified key *MSTRG.12678.2* and *MSTRG.1478.1* based on function and expression. Thus, these two lncRNA deserve further validation from the perspective of reverse genetics.

PRRSV has been reported to be an apoptotic-inductor virus both *in vivo* and *in vitro* (59). Previous studies have revealed that apoptosis was detected both in B- and T-cell areas of lymphoid organs and plays a role in the impairment of the host immune response during PRRS (60). We found that nuclear lncRNAs reversed by tartaric acid may regulate neighboring genes that play negative regulation of T cell apoptosis through *cis-*regulation, indicating that tartaric acid may play an important role in maintaining PAM’s immune cell apoptosis metabolism by stimulating lncRNAs. IL37 is a member of the IL1 family, which has been widely reported to play anti-inflammation roles in macrophages via regulating M1 and M2 polarization of macrophages (40). However, our knowledge of IL37 in PAM, especially PRRSV-infected PAM, remains unknown. LncRNA can exert *trans-*regulation by recruiting special proteins (61). Our data showed that both the sequences of nuclear TELs and TRLs were significantly enriched in the similar motif of IL37, which suggested that IL37 might play an important role in anti-inflammation caused by PRRS, and lncRNAs that bind to IL37 may be crucial for IL37 recruitment. Therefore, the selected six highly expressed lncRNAs with more than 3 IF37 binding sites thus warrant further investigation.

## 5 Conclusions

In summary, our data shed light on non-coding RNA in response to PRRSV and tylvalosin tartrates, and further functional validation of key genes might provide a solid foundation for future PRRSV prevention and control in molecular drug design and genetic improvement.

## Data Availability

The datasets presented in this study can be found in online repositories. The names of the repository/repositories and accession number(s) can be found below: PRJNA1169158 (SRA).
