# Supplementary material for: Analysis of whole transcriptome reveals the immune response to porcine reproductive and respiratory syndrome virus infection and tylvalosin tartrate treatment in the porcine alveolar macrophages
Source: Front Immunol. 2025 Jan 13;15:1506371. doi: 10.3389/fimmu.2024.1506371 (PMC11769836; doi:10.3389/fimmu.2024.1506371)
Supplement: Supplementary file 1 [file Table1.docx]

**Supplementary Table S1.** **The source code and hyperparameters of the neural network models**

1. Python source code of the neural network models in this study

import dgl

import torch.nn.functional as F

import torch

import torch as t

import dgl.nn.pytorch as dglnn

import torch.nn as nn

class GCN_attention(nn.Module):

def __init__(self, config, hidden_dim):

super(GCN_attention, self).__init__()

self.conv1 = dglnn.GraphConv(config.d, hidden_dim, norm='none', allow_zero_in_degree=True)

self.conv2 = dglnn.GraphConv(hidden_dim, hidden_dim, norm='none', allow_zero_in_degree=True)

self.multihead_attn = nn.MultiheadAttention(hidden_dim, num_heads=2)

self.classify = MLP(inSize=hidden_dim, outSize=config.n_classes)

self.moduleList = nn.ModuleList([self.conv1, self.conv2, self.multihead_attn, self.classify])

def forward(self, g, h):

# Apply graph convolution networks and activation functions

h = F.relu(self.conv1(g, h, edge_weight=g.edata['weight']))

h = F.relu(self.conv2(g, h, edge_weight=g.edata['weight']))

with g.local_scope():

g.ndata['h'] = h

# Use the average readout to get the graph representation

hg = dgl.mean_nodes(g, 'h')

# Applying two-head attention mechanism

attention_output, _ = self.multihead_attn(hg.unsqueeze(0), hg.unsqueeze(0), hg.unsqueeze(0), key_padding_mask=None, attn_mask=None)

hg = attention_output.squeeze(0)

return self.classify(hg.float())

class MLP(nn.Module):

def __init__(self, inSize, outSize, hiddenList=[], dropout=0.5, actFunc=nn.ReLU):

super(MLP, self).__init__()

layers = nn.Sequential()

for i,os in enumerate(hiddenList):

layers.add_module(str(i*2), nn.Linear(inSize, os))

layers.add_module(str(i*2+1), actFunc())

inSize = os

self.hiddenLayers = layers

self.dropout = nn.Dropout(p=dropout)

self.out = nn.Linear(inSize, outSize)

def forward(self, x):

x = self.hiddenLayers(x)

return self.out(self.dropout(x))

1. Hyperparameters used for training models

| Hyperparameter | Value |
| --- | --- |
| K value of k-mer | 5 |
| Node feature dimension | 128 |
| Hidden layer of the graph convolutional networks | 64 |
| Batch size | 8 |
| Epochs | 1000 |
| Learning rate | 0.003 |
| K of Fold | 10 |
| Early Stop | 100 |
