# Supplementary material for: Analysis of whole transcriptome reveals the immune response to porcine reproductive and respiratory syndrome virus infection and tylvalosin tartrate treatment in the porcine alveolar macrophages
Source: Front Immunol. 2025 Jan 13;15:1506371. doi: 10.3389/fimmu.2024.1506371 (PMC11769836; doi:10.3389/fimmu.2024.1506371)
Supplement: Supplementary file 8 [file Table8.docx]

**Supplementary Table S8. Primers Tsed in RT-qPCR**

| **NTmber** | **RNA Type** | **ID** | **Primer (forward/reverse)** |
| --- | --- | --- | --- |
| 1 | mRNA | *IFNG* | TCAGCTTTGCGTGACTTTGTG/ TGCTCCTTTGAATGGCCTGG |
| 2 | mRNA | *CD274* | ATGGTGGTGCCGACTACAAG/ GGTGATGGTGGTTTTGCCAC |
| 3 | mRNA | *IRF1* | TCCAGCCGAGATGCTAAGAG/ GCTGTGGTCATCAGGCAGAGT |
| 4 | mRNA | *IL21* | AGACACTGTGAGCAGTCAGC/ CCCTGCATTTGTGGGAGGTA |
| 5 | miRNA | *ssc-miR-30a-5p* | TGTAAACATCCTCGACTGGAAG |
| 6 | miRNA | *ssc-miR-218-5p* | TTGTGCTTGATCTAACCATGT |
| 7 | miRNA | *ssc-miR-218b* | TTGTGCTTGATCTAACCATGTG |
| 8 | lncRNA | *MSTRG.6608.1* | CCCTGCATTTGTGGGAGGTA/ CCCTGCATTTGTGGGAGGTA |
| 9 | lncRNA | *MSTRG.13889.3* | GTCTGCTTGTGGAATGCGTG/ AGGATAGGACACCAGCCTGT |
| 10 | lncRNA | *MSTRG.4312.1* | ACGGAGACCCAGCTTATGTG/ ACACAAACTGGAATGGGCCT |
