# Supplementary material for: Analysis of whole transcriptome reveals the immune response to porcine reproductive and respiratory syndrome virus infection and tylvalosin tartrate treatment in the porcine alveolar macrophages
Source: Front Immunol. 2025 Jan 13;15:1506371. doi: 10.3389/fimmu.2024.1506371 (PMC11769836; doi:10.3389/fimmu.2024.1506371)
Supplement: Supplementary file 9 [file Table9.docx]

Supplementary Material

# Supplementary Figures and Tables

For more information on Supplementary Material and for details on the different file types accepted, please see [here](https://www.frontiersin.org/guidelines/author-guidelines#supplementary-material).

## Supplementary Figures


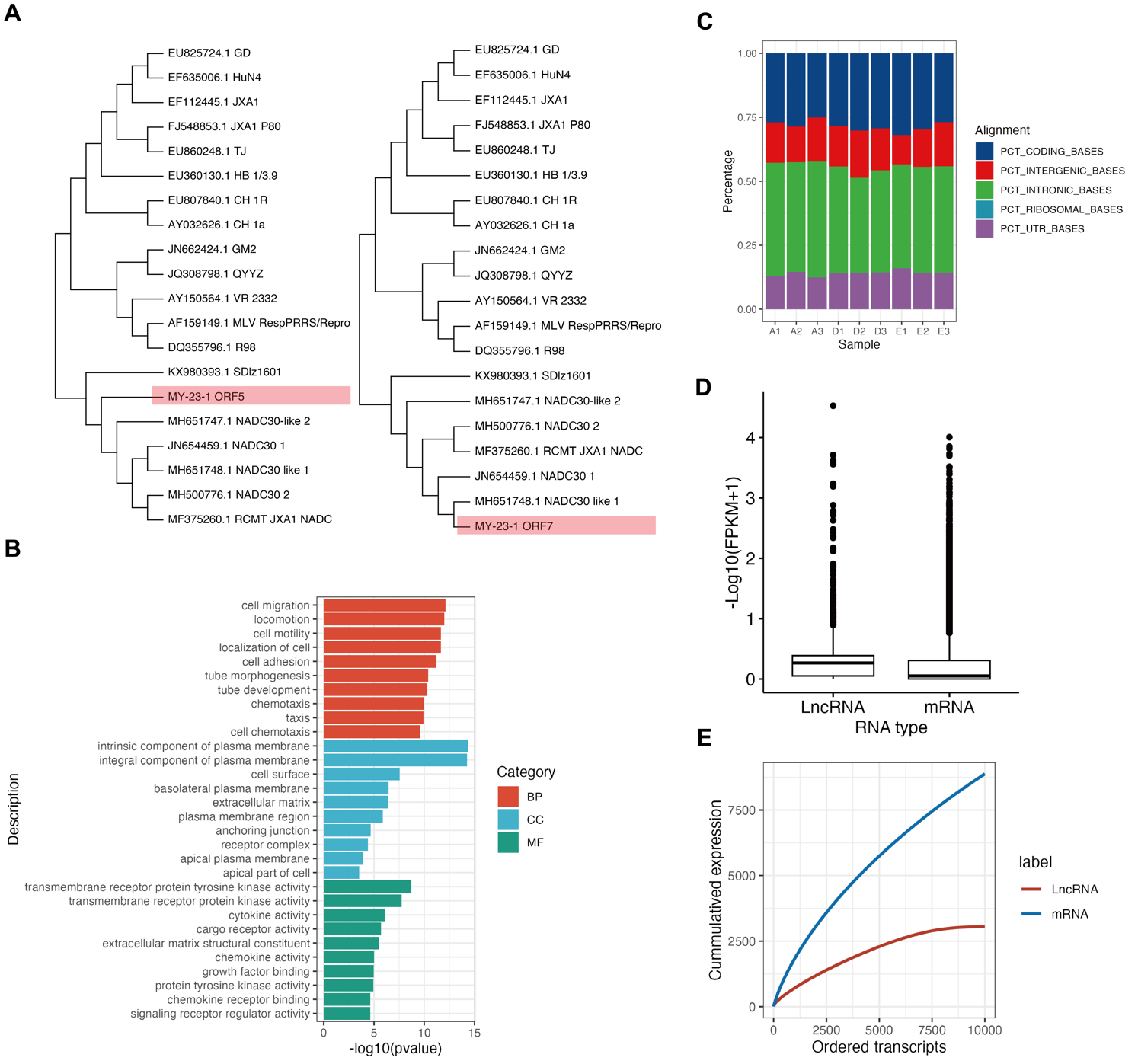


**Supplementary Figure S1.** De novo reconstruction of long non-coding RNAs of PAMs when PRRSV infection after administration of Tylvalosin tartrate. (A) Phylogenetic analysis of MY-23-1 strain using *Orf5* and *Orf7* genomic sequences. Left: *Orf5*; right: *Orf7*. (B) Gene Ontology enrichment analysis of differentially expressed genes (DEGs) between NCG and PCG. The BP, CC, and MF represent the category of biological processes, cellular components, and molecular functions, respectively. (C) Mapping sequencing reads to different genomic regions. (D) Expression comparison of mRNAs and IncRNAs. (E) Comparison of expression complexity of IncRNAs and mRNAs.


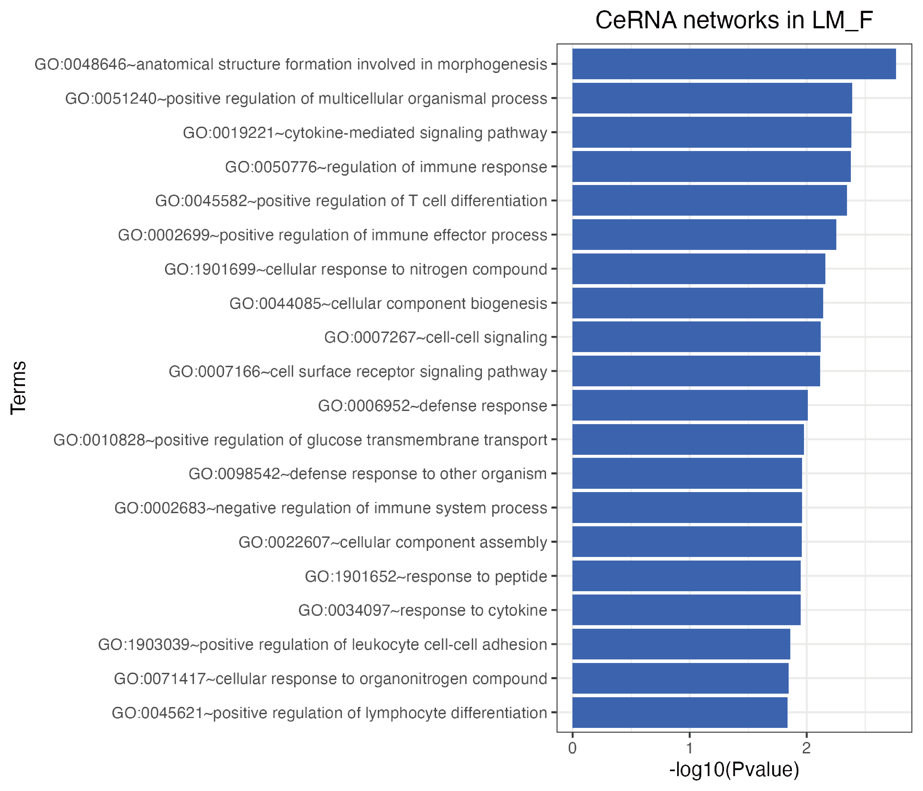


**Supplementary Figure S2.** GO enrichment of ceRNA networks in LM_E.


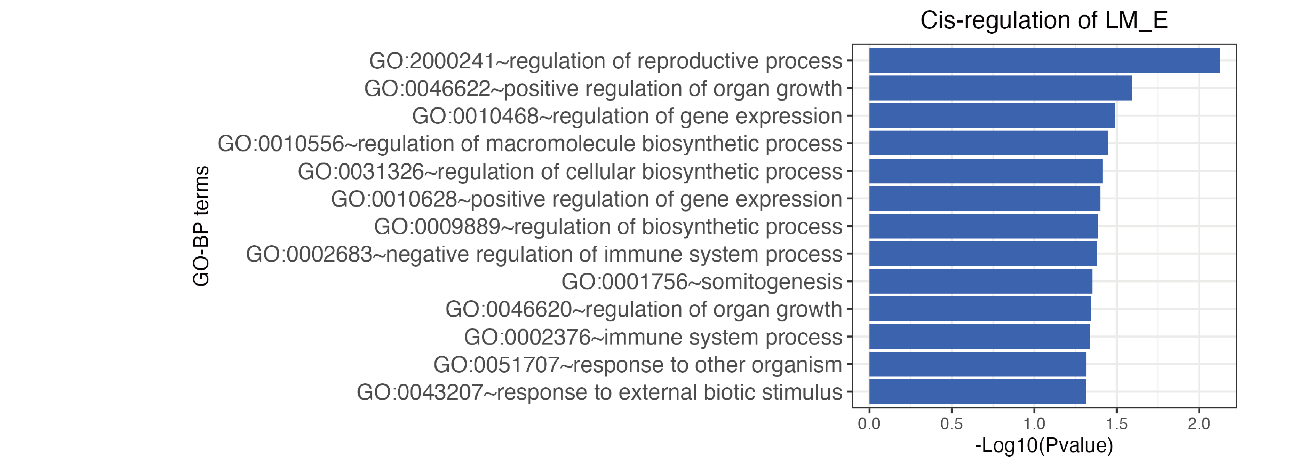


**Supplementary Figure S3.** GO enrichment of cis-regulation of lncRNAs in LM_E.

## Supplementary Tables

**Supplementary Table S1.** The source code and hyperparameters of the neural network models.

**Supplementary Table S2.** Summary of ssRNA-seq and miRNA-seq data.

**Supplementary Table S3.** Differentially expressed genes (DEGs).

**Supplementary Table S4.** Prediction of subcellular localization of differential lncRNAs and mRNAs.

**Supplementary Table S5.** Differentially expressed miRNAs (DEmiRNAs).

**Supplementary Table S6.** TEFLs involved lncRNA-miRNA-mRNA ceRNA networks in the cytoplasm.

**Supplementary Table S7.** TRFLs involved lncRNA-miRNA-mRNA ceRNA networks in the cytoplasm.

**Supplementary Table S8.** Primers used in RT-qPCR.
